# Diseases of the Skin

**Published:** 1904-01

**Authors:** 


Diseases of the Skin. A Manual for Students and Practitioners. By Joseph Grindon, Ph. D., M. D. (being of Lea's Series of Pocket Text-Books, series edited by Bern B. Gallaudet). Illustrated with 39 engravings. Lea Brothers & Co., Philadelphia and New York.

This work is a thoroughly modern and scientific discussion of its subject; of especial use to the student, but extensive and diversified enough for the general practitioner. Its poverty of illustrations is perhaps its greatest fault. The pictures shown are, however, well selected, typical, and of good quality. The text is ample and clear, making the work all that is claimed for it.

